# Structure and Dielectric Behavior of Yb_2_O_3_-MgO Co-Doped 0.92BaTiO_3_-0.08(Na_0.5_Bi_0.5_)TiO_3_ Ferroelectric Relaxor

**DOI:** 10.3390/ma14226802

**Published:** 2021-11-11

**Authors:** Xiao-Hu Ren, Dong-Yun Gui

**Affiliations:** College of Materials Science and Engineering, Xi′an University of Architecture and Technology, Xi′an 710054, China; renxiaohu@xauat.edu.cn

**Keywords:** ferroelectric relaxor, core–shell structure, Raman spectra, TEM, dielectric properties

## Abstract

Dielectric properties and structure of 0.015Yb_2_O_3_-*x*MgO doped 0.92BaTiO_3_-0.08(Na_0.5_Bi_0.5_)TiO_3_ ceramics with *x* = 0.0–0.025 have been investigated. As Yb_2_O_3_-MgO was added into the BT-NBT, the phase changes from tetragonal to pseudo-cubic, with the tetragonality *c*/*a* decreases from 1.011 to 1.008 and XRD peaks broadened. The combined study of XRD and TEM image revealed a formation of core–shell structure in grains with core of 400–600 nm and the shell of a thickness 60–200 nm. There is a slowly phase transition against temperature from the variable temperature Raman analysis. The ferroelectric relaxor peak of BT-NBT decreases from ~4000 to ~2000 and a new broad dielectric peak with an equivalent maximum (*ε*_r_′~2300) appears in the temperature dependent dielectric constant curve (*ε_r_*′-*T*), which produces a flat *ε_r_*′-*T* curve. Sample 0.92BaTiO_3_-0.08(Na_0.5_Bi_0.5_)TiO_3_-0.015Yb_2_O_3_-0.005 MgO and 0.92BaTiO_3_-0.08(Na_0.5_Bi_0.5_)TiO_3_-0.015Yb_2_O_3_-0.01MgO give a *ε*_r_′ variation within ±14% and ±10% in 20–165 °C. The core–shell microstructure should take account for the flattened *ε*_r_′–*T* behavior of these samples.

## 1. Introduction

BaTiO_3_-based (BT) materials with large dielectric constant (*ε_r_*′) are widely used in electric components and devices [1], such as high permittivity ceramic capacitors, piezoelectric ceramic transducers, and sensors. Generally, a key parameter of dielectric materials in applications is the permittivity (*ε_r_*′) and its temperature stability. For example, in multi-layer ceramic capacitors (MLCC), ideal materials should give a high *ε_r_*′ and a flat *ε_r_*′-*T* curve (relaxed) in the operation temperature range. The international EIA of Electronic Industries Association USA (Electronic Industries Association) has proposed a MLCC standard [2] on the rate of change of capacitance against temperature (ΔC/C_20°C_), in which an X8R type MLCC is ΔC/C_20°C_ in ± 15% from −55 to 150 °C [3]. As a ferroelectric material, the *ε_r_*′ of BaTiO_3_ (BT for short) is high (~10^4^) at the vicinity of phase transition temperature (*T*_c_~393 K) and drops significantly at temperature away from *T*_c_, which makes it not immediately suitable for capacitor applications. There are many researches focused on the improvement of the *ε_r_*′-*T* curve by the additions of some oxides into BaTiO_3_, such as Nb_2_O_5_, MgO, Co_3_O_4_, MnCO_3_ [4], and many BT-based ferroelectric relaxors reported. Among which, MgO shows the significant effects to the dielectric behavior and microstructure of BT ceramics [5,6,7]. For example, a suitable MgO-Nb_2_O_5_-Na_0.5_Bi_0.5_TiO_3_-BT relaxor ceramic can achieves an EIA-X9R standard, showing ΔC/C_20°C_ = −1.8, 1.8, and −5.69% at −55, 125, and 200 °C, respectively [6].

On the relaxation mechanism of BT-based ferroelectric relaxors, the microstructure, chemical inhomogeneity and dielectric properties were focused [5,7,8,9,10,11,12,13]. It is found that BT-based relaxors show very subtle “core–shell” grain microstructures with different chemistry compositions, which should be the origin of the relaxed dielectric behaviors. For example, in MgO-Y_2_O_3_-BT ceramics, the core of grains is mainly BT ferroelectric domains, the Y^3+^ is mainly located in the shell of grains and Mg^2+^ is mainly at grain boundaries. Thus, in order to obtain a flat *ε_r_*′-*T* curve for applications, the control of core–shell microstructure in BaTiO_3_-based dielectric ceramic is an effective way [11,14,15]. (Na_0.5_Bi_0.5_)TiO_3_ (NBT for short) with a perovskite structure is one of the most important ferroelectric material, which shows a high Curie temperature *T*_c_ ~ 593 K. NBT is widely used for the modification of BT ceramics in recent researches to move the *T*_c_ to a high temperature and obtain a broad *ε_r_*′-*T* curve [14,15,16,17,18,19]. However, the *ε_r_*′-*T* curve of BT-NBT still shows significant peak behavior in our experience, which make it not suitable in applications. Thus, a further improvement on the *ε_r_*′-*T* relaxing behavior is needed.

In this work, we selected 0.92BaTiO_3_-0.08(Na_0.5_Bi_0.5_)TiO_3_ (abbreviated as BT-NBT) systems as matrix and Yb_2_O_3_-MgO as modifications to study the microstructure/local structure and dielectric properties, which has not been studied. We prepared Yb_2_O_3_-MgO doped BT-NBT ceramic samples and investigated the XRD, Raman, and dielectric behaviors.

## 2. Materials and Methods

0.92BaTiO_3_-0.08(Na_0.5_Bi_0.5_)TiO_3_-0.015Yb_2_O_3_-*x*MgO (BT-NBT-YM for short) were prepared by high temperature solid-state reaction method. The raw materials selected are BaTiO_3_, Bi_2_O_3_, Na_2_CO_3_, TiO_2_, Yb_2_O_3_, and MgO with the purity > 99% (Sinopharm, Beijing, China). (Na_0.5_Bi_0.5_)TiO_3_ powders was prepared from Bi_2_O_3_, Na_2_CO_3_, and TiO_2_. Stoichiometric materials were mixed and calcined at 800 °C for 2 h. After cooled, the product was ground (labeled as NBT). Then, the NBT and BaTiO_3_ powders were weighed and mixed by ball milling for 24 h. The slurry was dried and calcined at 900 °C for 2 h. The cooled product was ground, added with 1.5 mol% Yb_2_O_3_ and *x* mol% MgO (YM for short) and mixed by ball-milled method for 24 h. The slurries were dried and pressed into pellets at ~200 MPa (with 5% PVA as binder). These pellets were sintered at 1240~1270 °C for 3 h (optimized condition) to obtain the final ceramic samples. All the ceramics were carefully polished for further measurements.

The average crystal structure of samples was analyzed by an X-ray diffractometer (X′Pert Pro Panalytical, Almelo, The Netherlands) with scanning rate 1°/min and Cu-Kα radiation (40 kV × 40 mA). The local structure of these samples was analyzed using Raman spectra, which were collected on a Renishaw Invia Raman Microscope spectrometer (Charfield, UK). The spectrometer was equipped with a diffraction grating of 1800 grooves/mm and a confocal microscope (100× objective) to ensure a spectrum resolution of ~2 cm^−1^. The spectra were collected in backscattering geometry excited by an argon laser at 514.5 nm. A TMS 94 module was used for temperature controlling from 30–200 °C during the measurements. The microstructures of ceramics were investigated by a transmission electron microscopy (TEM, JEOL, Tokyo, Japan), and the samples for the TEM measurements were prepared by ion-beam thinning. Temperature-dependent dielectric parameters were measured using an LCR meter (HP E4980A, Palo Alto, CA, USA) from 20 to 250 °C. For the dielectric measurements, the polished samples were coated with silver paste as electrodes. 

## 3. Results and Discussion

### 3.1. XRD and the Phase

**Figure 1:**
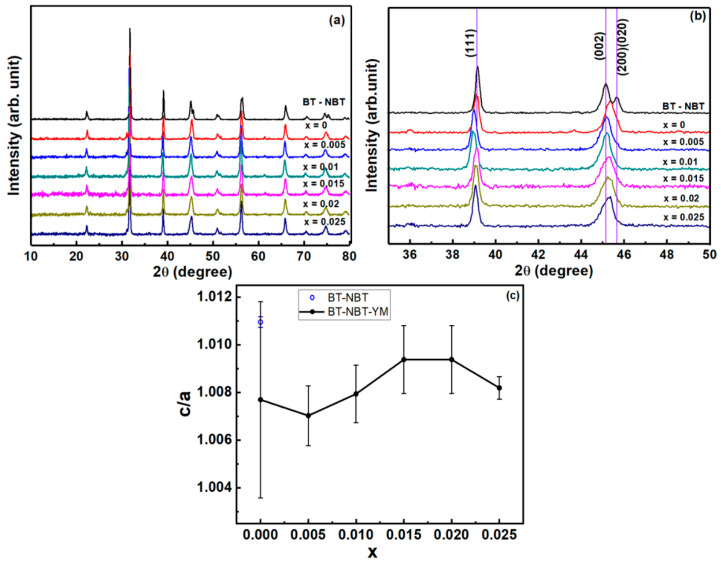
(**a**) XRD patterns of BT-NBT and BT-NBT-YM samples, (**b**) the magnified data for BT-NBT-YM samples, (**c**) the tetragonality *c*/*a* of samples from Pawley refinements.

The XRD patterns of BT-NBT-YM samples at room-temperature are shown in Figure 1. Generally, the symmetry of BT-ceramics is tetragonal or cubic/pseudo-cubic nearby room temperature. The difference between them is the relation of cell parameters *a*, *b* and *c* (tetragonality *c*/*a* ratio), which gives an “overlapped” peak for the (*n*00) (0*n*0) (00*n*) reflections with the same *n* in cubic phase but two peaks [(*n*00) (0*n*0) peak and (00*n*) peak] in the tetragonal one. The *c*/*a* = 1 for an ideal cubic phase and *c*/*a* > 1 for tetragonal BT phase. However, for *c*/*a* ≈ 1, it is hard to recognize between tetragonal and cubic ones, which are considered as a pseudo-cubic phase. The undoped BT-NBT ceramics give an obvious tetragonal perovskite structure with separated (200) (020) peak and (002) peak (Figure 1b). The tetragonality *c*/*a* of BT-NBT from Pawley refinements (Figure 1c) is 1.011. As YM was added into the system, the (200) (020) peak and (002) peak shift and merge together and the XRD peaks become broadened. Thus, the phase of the BT-NBT system transforms from tetragonal to pseudo-cubic with the “average” tetragonality *c*/*a* ≈ 1.008 (Figure 1c). The tetragonality is supposed to dominates the intrinsic dielectric behavior. In our experience, for samples with similar microstructures, the higher tetragonality, the narrower *ε*_r_′–T relaxing peak. Meanwhile, the size of crystallization domains decreases or more strain is produced, which causes the broadening of the XRD peaks. Hsiang et al. [13] proposed that this agrees with the formation of the core–shell structure in the dielectric grains. Theoretically, the phase and crystal structure of the core and shell parts should be different. However, the crystal structure between them should be quite similar. Considering the effect of peak broadening, it should be hard to distinguish their XRD reflections. Thus, there is no obvious two-phase reflections observed in the XRD measurements.

### 3.2. Raman Spectra

**Figure 2:**
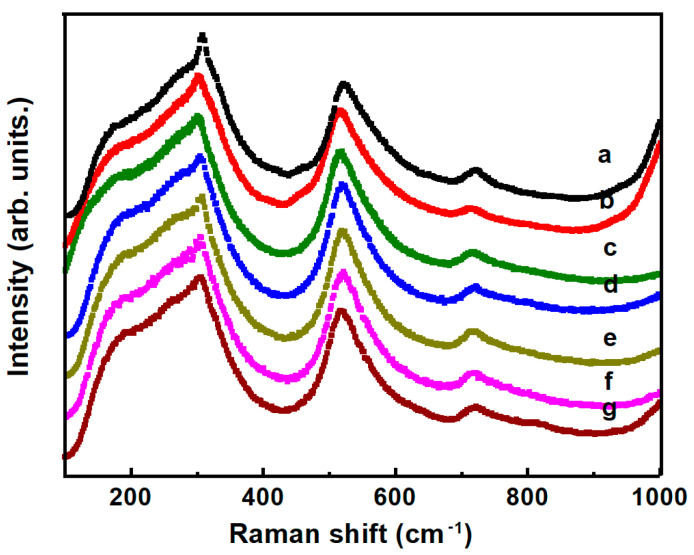
Raman spectra of pure BT-NBT ceramics and Yb-Mg codoped BT-NBT ceramics at room temperature, where (a) undoped BT-NBT; (b) *x* = 0; (c) *x* = 0.005; (d) *x* = 0.01; (e) *x* = 0.015; (f) *x* = 0.02; (g) *x* = 0.025.

The room temperature depolarized Raman spectra of BT-NBT and BT-NBT-YM ceramics are plotted in Figure 2. All the spectra show similar characteristic, which means a similar symmetry of the samples. According to the selection rule, BT with cubic symmetry does not have first-order Raman active mode, whilst BT with tetragonal symmetry (*P*4*mm*, #99) has the eight first-order Raman active modes (3*A*_1_ + *B*_1_ + 4*E*). The broad modes locate at ~118, 152, 188, 289, 509, and 737 cm^−1^ mean a local symmetry fluctuation in the cubic background (pseudo-cubic). Refer to results of Ba(Zr*_x_*Ti_1−*x*_)O_3_ [20] and (Ba_1−*x*_Sr*_x_*)TiO_3_ ceramics [21], we assigned the four obvious Raman modes at 170, 307, 521, and 718 cm^−1^ to *A*_1_(TO_1_), *E*(TO_2_), *A*_1_(TO_3_), and *A*_1_(LO_3_) modes, respectively.

**Figure 3:**
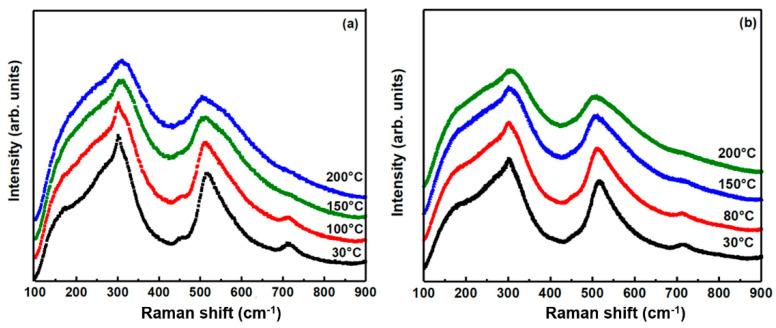
(**a**) Raman spectra of undoped BT-NBT ceramics at different temperatures; (**b**) Raman spectra of BT-NBT-YM (*x* = 0.015) ceramics at different temperatures.

The temperature-dependent Raman spectra of BT-NBT and BT-NBT-YM (*x* = 0.015) ceramics are plotted in Figure 3. Generally, all the Raman peaks are broadened during the heating. The *E*(TO_2_) mode at ~307 cm^−1^ is considered to be the key modes associates with the tetragonal-cubic phase transition [22] in complex-perovskites and a decreasing intensity is observed as the temperature increases. Thus, a slow phase transition (from low symmetry to high symmetry) occurs with the increase of temperature. The Raman spectra of BT-NBT and BT-NBT-YM are quite similar, which means a similar phase transition behavior between them. This agrees with the similar ferroelectric relaxing behavior observed in the followed sections.

### 3.3. TEM Analysis

**Figure 4:**
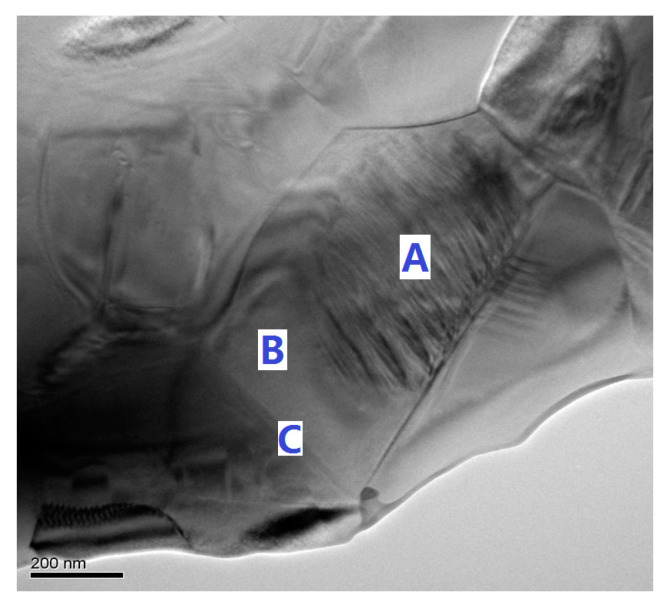
TEM image of BT-NBT-YM ceramic (*x* = 0.015). A, B, C: the core, shell region of a grain and grain boundary.

Generally, a composite of doped-BaTiO_3_ with a low Curie temperature (shell) and undoped-BaTiO_3_ with a high Curie temperature (core) is known to show a flat permittivity over a wide temperature. To confirm the “core–shell” structure in the BT-NBT-YM samples, we investigated the TEM image of a selected sample (*x* = 0.015). A typical TEM image of the ceramic is shown in Figure 4.

Obvious “core–shell” grains and grain boundaries can be recognized. There are ferroelectric domain patterns (A) in the core region of grain surrounded by paraelectric shell region (B) of grain and grain boundary (C). For the selected grain, the size of the core (A) is 400–600 nm and the thickness of the shell is 60–200 nm. The crystal structure of the core and shell region should be tetragonal phase and cubic/pseudo-cubic phase, respectively. The crystal structure between them should be quite similar and the small size of core region and shell region causes the broadening of their XRD peaks, which makes the character peaks of them hard to be distinguished in the overlapped XRD patterns.

### 3.4. Dielectric Parameters

**Figure 5:**
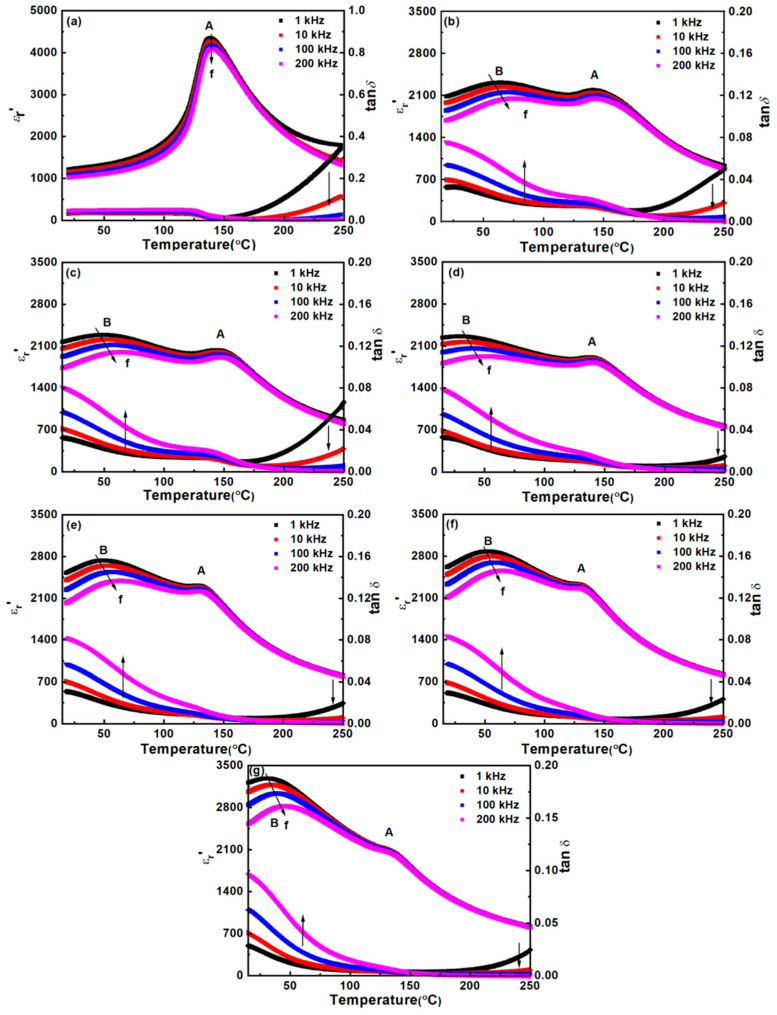
Temperature-dependent permittivity (*ε*_r_′) and the dielectric loss (tan*δ*) of the BT-NBT-YM samples at different frequencies. (**a**) BT-NBT, (**b**) *x* = 0, (**c**) *x* = 0.005, (**d**) *x* = 0.01, (**e**) *x* = 0.015, (**f**) *x* = 0.02, and (**g**) *x* = 0.025; A, B indicates the different peaks of *ε*_r_′–*T. f*: frequency.

The temperature-dependent real permittivity (*ε*_r_′) and the dielectric loss (tan*δ*) of the BT-NBT-YM samples are shown in Figure 5, and the temperature-dependent complex permittivity (*ε*_r_* = *ε*_r_′ − j*ε*_r_″) of samples are shown in Figure S1 in the supplementary information (SI). For the BT-NBT sample (Figure 5a and Figure S1a), it shows typical ferroelectric relaxor behavior. There is a broad peak at ~140 °C both in *ε*_r_′–*T* (labelled as “A” in Figure 5a) and *ε*_r_″–*T* curve. With the increase of frequency, the real *ε*_r_′–*T* peak slightly decreases but the imaginary *ε*_r_″–*T* peak slightly increases. The loss tan*δ* increases with the increase of frequency at lower *T* (<180 °C) but the decreases with the increase of frequency at higher *T* (>180 °C). The dielectric loss at higher and lower temperature here is named as tan*δ*′ and tan*δ″*, respectively. The tan*δ″* is almost independent with *T* but the tan*δ*′ is greatly influenced by temperature *T*.

As 1.5 mol % Yb_2_O_3_ was added into the BT-NBT (BT-NBT-Yb_2_O_3_), the peak “A” decreases from ~4000 to ~2000 and a new broad dielectric peak (labelled as “B” in Figure 5b) with an equivalent maximum (*ε*_r_′~2300) appears in *ε*_r_′–*T* curve. The two-peak like behavior is also observed in the *ε*_r_″–*T* curve (Figure S1b). Similarly, the real *ε*_r_′–*T* peaks (both A and B) slightly decrease but the imaginary *ε*_r_″–*T* peaks slightly increase with the increase of frequency, which means that both are ferroelectric relaxor responses. As MgO was added to the Yb_2_O_3_-BT-NBT forming BT-NBT-YM, the dielectric behavior is similar to that of Yb_2_O_3_-BT-NBT system. With the increase of *x* (MgO), the dielectric peak “A” almost keeps unchanged (~2200). However, the peak “B” shifts to lower temperature for *x* ≤ 0.01 with the position almost unchanged but the maximum increases for *x* ≥ 0.015, e.g., *ε*_r_′ = 2300 at 325 K for *x* = 0.005, *ε*_r_′ = 1900 at 323 K for *x* = 0.01, and *ε*_r_′ = 2500 at 330 K for *x* = 0.02, at 200 kHz. Thus, YM significantly improves the ferroelectric relaxing performance. The dielectric loss especially tan*δ*′ decreases at *x* ≤ 0.01 but keeps almost unchanged at *x* ≥ 0.015. Sample 0.92BaTiO_3_-0.08(Na_0.5_Bi_0.5_)TiO_3_-0.015Yb_2_O_3_-0.005MgO and 0.92BaTiO_3_-0.08(Na_0.5_Bi_0.5_)TiO_3_-0.015Yb_2_O_3_-0.01MgO give the best ferroelectric relaxor behavior, which give an *ε*_r_′ variation within ± 14% and ± 10% in 20–165 °C at 200 kHz.

Refer to the studies reported on BT-based dielectric materials and our XRD, TEM, and Raman results, the observed *ε*_r_′–*T* behavior of BT-NBT-YM here should be caused by the “core–shell” microstructure. The broad permittivity temperature characteristics should be originated from the compositional inhomogeneity in grains, the dielectric behavior should be a superposition of the characteristics of ferroelectric grain core (peak A), paraelectric grain shell (peak B) and concentration gradient region between them according to ref. [23], Jeon et al., also proposed a solution–precipitation mechanism to explain the microstructure formation process, in which the shell is a result of the precipitation of dopants (Yb_2_O_3_ and MgO in this study) from the liquid matrix containing BT-NBT during sintering, and the core region is essentially large BT-NBT grains. Though it is not distinguished in our XRD analysis for BT-NBT-YM samples due to the sensitivity and peak broadening, the samples should be in a complex subtle inhomogeneous microstructure and the tetragonality of core should be larger than that of the shell. Thus, for the observed *ε*_r_′–*T* behaviors, the peak A should be dominated by the ferroelectric core (mainly BT-NBT, region A in Figure 4), which is less affected by the increasing of MgO. The peak B should be dominated by the shell (BT-NBT with more Yb_2_O_3_ and MgO, solid solution or composite, region B in Figure 4), which is significantly affected by the MgO-Yb_2_O_3_. As the shell is similar to the core but with a slightly different composition to that of the core, it gives a transition *ε*_r_′-*T* peaks similar to that of the core but the peak at a lower T than that of the core (peak A). Both theoretical and experimental details on the mechanism of the ferroelectric relaxation of BT-NBT-YM need to be investigated in our future studies.

## 4. Conclusions

1.5% Yb_2_O_3_-*x* mol MgO doped 0.92BaTiO_3_-0.08(Na_0.5_Bi_0.5_)TiO_3_ ceramics with *x* = 0.0–0.025 are prepared by the solid state reaction method. The ceramics were analyzed by x-ray diffractions, dielectric measurements, TEM and Raman spectra. As Yb_2_O_3_-MgO added, the phase changes from tetragonal to pseudo-cubic, with the tetragonality *c*/*a* decreases from 1.011 to 1.008 and XRD peaks broadened obviously. There is a slow phase transition with the temperature changed from variable temperature Raman measurements and core–shell grain microstructures with core of 400–600 nm and a shell of 60–200 nm thickness is observed. A new broad peak in temperature dependent dielectric constant curve is also observed as Yb_2_O_3_–MgO added into the 0.92BaTiO_3_-0.08(Na_0.5_Bi_0.5_)TiO_3_, which improves the overall relaxor behavior. Sample 0.92BaTiO_3_-0.08(Na_0.5_Bi_0.5_)TiO_3_-0.015Yb_2_O_3_-0.005MgO and 0.92BaTiO_3_-0.08(Na_0.5_Bi_0.5_)TiO_3_-0.015Yb_2_O_3_-0.01MgO give the best ferroelectric relaxor behavior, which give an *ε*_r_′ variation within ± 14% and ± 10% in 20–165 °C.

## Supplementary Materials

The following are available online at https://www.mdpi.com/article/10.3390/ma14226802/s1, Figure S1: Temperature-dependent permittivity (*ε**_r_* = ε_r_′ − jε_r_″*) of the BT-NBT-YM samples at different frequencies.
