# Supplementary material for: Structure and Dielectric Behavior of Yb2O3-MgO Co-Doped 0.92BaTiO3-0.08(Na0.5Bi0.5)TiO3 Ferroelectric Relaxor
Source: Materials (Basel). 2021 Nov 11;14(22):6802. doi: 10.3390/ma14226802 (PMC8618925; doi:10.3390/ma14226802)
Supplement: Supplementary file 1 [file materials-14-06802-s001.zip › materials-1440809-supplementary.pdf]

materials

Structure and Dielectric Behavior of Yb_2O_3-MgO Co-Doped $0.92BaTiO_3$-$0.08(Na_{0.5}Bi_{0.5})TiO_3$ Ferroelectric Relaxor

Xiao-Hu Ren and Dong-Yun Gui *

College of Materials Science and Engineering, Xi'an University of Architecture and Technology, Xi'an 710054, Shaanxi, China; renxiaohu@xauat.edu.cn (X.-H.R.)
* Correspondence: guidy@xauat.edu.cn

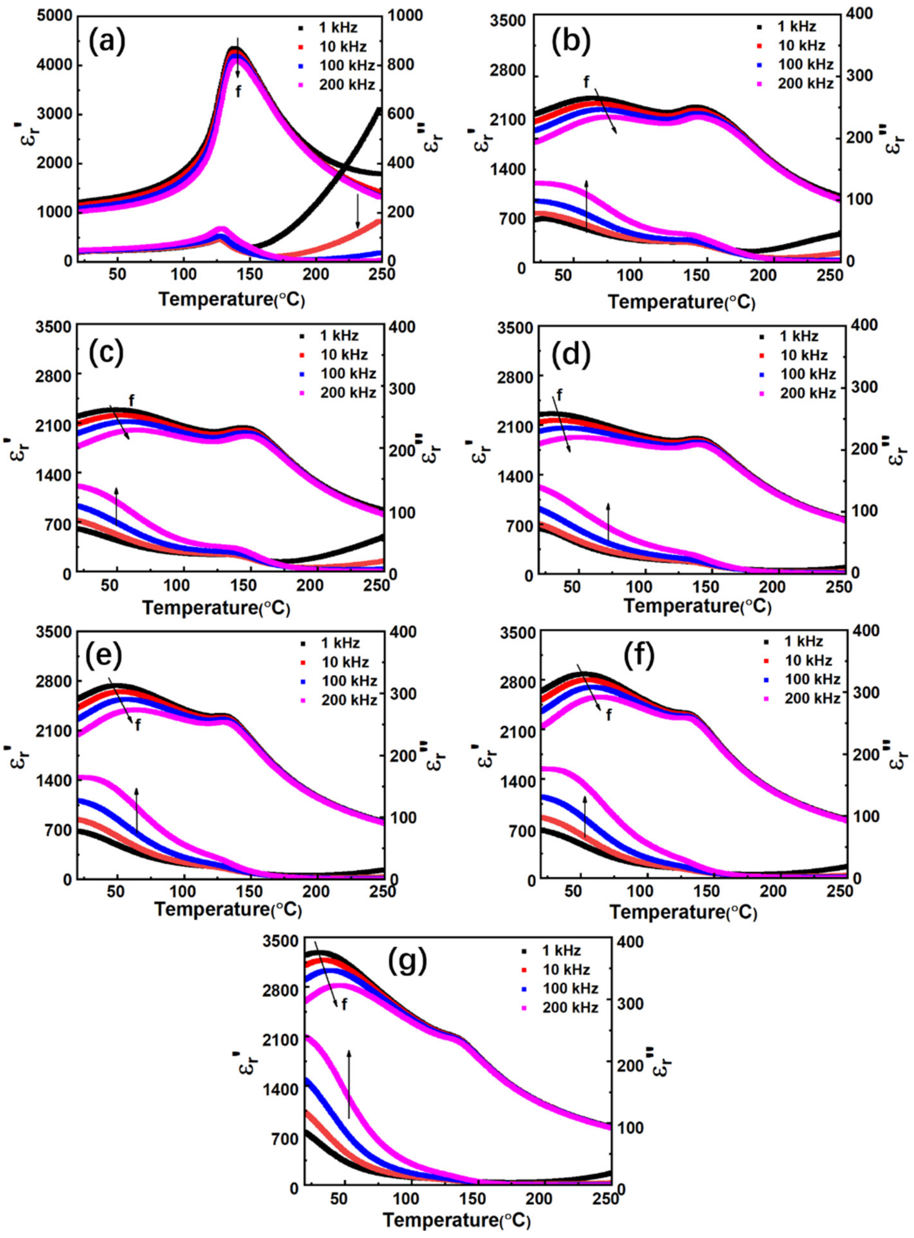

Figure S1. Temperature-dependent permittivity ($\varepsilon_r^* = \varepsilon_r' - j\varepsilon_r''$) of the BT-NBT-YM samples at different frequencies. (a) BT-NBT, (b) x = 0, (c) x = 0.005, (d) x = 0.01, (e) x = 0.015, (f) x = 0.02, (g) x = 0.025.

Citation: Ren, X.-H.; Gui, D.-Y.; Structure and Dielectric Behavior of Yb_2O_3-MgO Co-Doped $0.92BaTiO_3$-$0.08(Na_{0.5}Bi_{0.5})TiO_3$ Ferroelectric Relaxor. *Materials* **2021**, *14*, 6802. https://doi.org/10.3390/ma14226802

Academic Editor: Jan Dec

Received: 13 October 2021
Accepted: 5 November 2021
Published: 11 October 2021

Publisher's Note: MDPI stays neutral with regard to jurisdictional claims in published maps and institutional affiliations.

Copyright: © 2021 by the authors. Submitted for possible open access publication under the terms and conditions of the Creative Commons Attribution (CC BY) license (https://creativecommons.org/licenses/by/4.0/).
